# Ion Exchange Membrane-like Deposited Electrodes for Capacitive De-Ionization: Performance Evaluation and Mechanism Study

**DOI:** 10.3390/membranes15110338

**Published:** 2025-11-11

**Authors:** Siyue Xue, Chengyi Wang, Tianxiao Leng, Chenglin Zhang, Long-Fei Ren, Jiahui Shao

**Affiliations:** 1 State Key Laboratory of Green Papermaking and Resource Recycling, School of Environmental Science and Engineering, Shanghai Jiao Tong University, Shanghai 200240, China; xsybearcat18@sjtu.edu.cn (S.X.);; 2 National Observation and Research Station of Erhai Lake Ecosystem in Yunnan, Yunnan Dali Research Institute, Shanghai Jiao Tong University, Dali 671006, China; 3 Yazhou Bay Institute of Deepsea Science and Technology, Hainan Research Institute, Shanghai Jiao Tong University, Sanya 572025, China; 4 Chongqing Research Institute, Shanghai Jiao Tong University, Chongqing 401151, China

**Keywords:** membrane capacitive deionization, PEI, salt adsorption capacity, long-term stability

## Abstract

Capacitive de-ionization (CDI) holds great promise for water desalination, while the widely used activated carbon (AC) electrodes suffer from a low salt adsorption capacity (SAC) and poor long-term stability due to the co-ion effect and electrode oxidation. Inspired by membrane-based CDI, we deposited polyethyleneimine (PEI), an ion exchange polymer with positive charge and ion selectivity, onto an AC electrode to serve as an anode for addressing these issues. Firstly, compared to traditional AC and commercial AEM-AC, the PEI-doped AC (PDAC) anode delivered a superior SAC of 36.3 mg/g, as the positively charged PEI enhanced electrostatic attraction, suppressed the co-ion effect, and offered extra sites. However, it showed poor cycling stability with 77.1% retention, owing to mass loss and anode oxidation. We further developed an electrode coated with a PEI-based membrane (PMAC), which exhibited a balanced performance with a high SAC of 33.4 mg/g and significantly improved long-term retention of 97.5%. The hydrophilic PEI membrane, strongly adhered to the AC surface, shortened the ion diffusion resistance and effectively prolonged the electrode lifespan. A systematic comparison between AC, AEM-AC, PDAC, and PMAC revealed the mechanism for PMAC’s notable enhancement. These findings establish a framework for designing novel CDI electrodes and advancing sustainable water desalination.

## 1. Introduction

With the incessant growth of the global population and the accelerating pace of industrialization and urbanization, the demand for water resources is witnessing a sharp upward trajectory [1]. According to relevant statistical data, over 40% of the world’s population is currently confronted with varying degrees of water scarcity, and numerous regions are unable to ensure their residents’ access to an adequate and safe supply of freshwater [2]. One of the feasible ways to meet the freshwater demand is to develop brackish water treatment technology, eliminating salt and other impurities from brackish water through a series of physical, chemical, or biological techniques, thereby transforming it into freshwater that can be utilized for human production and daily life [3]. Nowadays, the commonly employed methods include multi-stage flash evaporation, electrodialysis, vapor compression, multiple effect distillation, and membrane separation technology [4]. Although these technologies have played a positive role in alleviating water scarcity, there remain challenges such as high energy consumption, expensive capital cost, and secondary pollution, which need further improvement and optimization [5].

Capacitive de-ionization (CDI) is an emerging electrochemical desalination technology that removes ions from brackish water by adsorbing them into the electrical double layer at the electrode surface [6,7]. Compared with traditional desalination technologies, CDI boasts several advantages, including lower energy consumption, simpler operation, less environmental impact, and relatively straightforward equipment maintenance [8]. The characteristics of CDI make it highly suitable for coupling with other water treatment technologies, such as ultrafiltration, as an alternative or pretreatment solution to reverse osmosis for low-salinity brackish water desalination and brackish water reuse [9]. Moreover, with the continuous advancement of materials science and other related fields, key components such as electrode materials employed in CDI are being continuously optimized, and its desalination efficiency and performance are anticipated to be further enhanced. CDI demonstrates distinctive merits when treating brackish water with low and moderate concentrations (200~1000 mg/L), while it exhibits poor desalination performance when treating solutions with high concentration (with a salt concentration in excess of 1000 mg/L), owing to the co-ion interference [10,11]. This occurs as ions of the same polarity are continuously adsorbed and desorbed from the electrode pores, reducing the amount of counter-ions that can be adsorbed from the feed, thereby decreasing the charge efficiency and adsorption capacity [12]. In addition to the co-ion effect, CDI electrodes also suffer from parasitic reactions, especially anode oxidation, which leads to the deterioration of electrode properties and desalination performance over long-term operation.

Recently, membrane capacitive de-ionization (MCDI), a modified version of traditional CDI, has been attracting more and more attention as a means to mitigate the above-mentioned limitations, which was first reported by Lee et al. in 2006 [13]. In MCDI, there is an anion exchange membrane (AEM) placed in front of the anode and a cation exchange membrane (CEM) positioned over the cathode [14]. The presence of charged groups in IEMs results in the selective transport of ions of opposite charge (counter-ions) and blocks the transport of ions of the same charge (co-ions), thus inhibiting the co-ion interference and enhancing the salt removal [15]. Moreover, these membranes serve as a protective layer to safeguard the electrode surface from harmful Faradaic reactions as a result of contact with saline water, thereby preventing anode oxidation and retaining stable cycling performance [16]. However, the membrane’s physical attachment obstructs influent flow and occupies adsorption sites, and its thickness increases ionic diffusion resistance, thereby limiting mass transfer [17].

Inspired by membranes, incorporating ion exchange polymers directly into electrode premixtures and constructing a composite electrode is regarded as an ideal method [18]. A novel CDI electrode doped with a crosslinked cation exchange polymer was designed by Kim and Choi [19], and results showed that the desalination performance was increased by 27–56%. By endowing the electrode pores with an ion-selective layer, the selective transport of counter-ions from the bulk solution to the electrode surface was enhanced. However, the weak bonding (e.g., hydrogen bonds) between ion exchange polymers and the carbon electrode causes mass loss and poor cycling stability during long-term operation [20]. Alternatively, directly depositing an ion exchange layer onto the electrode surface to form a thin layer with low electrical resistance on the outermost surface has gained intensive attention. Recently, an excellent desalination capacity and stable cycling performance were reported by Gao et al. using a polymer-deposited carbon cloth as an anode, and they attributed the improvement to the minimization of the co-ion effect and carbon oxidation by the polymer [21]. Unfortunately, the obtained electrodes prepared by this method usually suffer from damage to the pore structure, in turn decreasing the available surface area for ion adsorption [22]. Furthermore, the strong adhesion of the polymer membrane to the base electrode remains a challenge, as the membrane might undergo shear failure or detachment under the continuous influence of erosion. Therefore, the casting method for preparing membrane-deposited electrodes requires further optimization. More critically, while introducing ion exchange membranes or polymers can enhance CDI performance, the underlying mechanisms and a systematic evaluation of these improvements remain insufficiently explored.

The cationic polyethyleneimine (PEI) exhibits excellent film-forming ability and, upon protonation in water, strongly adsorbs anions. Its mechanical strength can be enhanced through chemical crosslinking, making it suitable for practical CDI use. Hence, two types of CDI anodes were fabricated: one by doping crosslinked PEI with activated carbon (PDAC), and the other by grafting a crosslinked PVA/PEI membrane onto a sheet AC electrode (PMAC). For comparison, a single AEM was placed in front of the AC anode to construct MCDI. The desalination performance of AC, AEM-AC, PDAC, and PMAC in terms of salt adsorption capacity (SAC), charge efficiency (CE), and cycling stability was systematically evaluated. Potential mechanisms for performance were explored through relevant characterization techniques and pH changes. Effects of parameters including voltage, initial salt concentration, flow rate, and co-existing ions were investigated to optimize CDI performance. Furthermore, PMAC maintained superior performance under varying operational conditions (voltage, salt concentration, and flow rate) and exhibited remarkable selectivity for Cl^−^ over coexisting ions. These advantages are attributed to its strongly adherent charged PVA/PEI membrane, which shortened ion transport pathways, suppressed co-ion effects and anode oxidation, and enhanced mechanical strength. This finding provides a feasible PEI-based modification strategy for developing high-performance CDI anodes in practical desalination applications.

## 2. Materials and Methods

### 2.1. Materials

Commercial ACs and AEMs were purchased from Yihuan Carbon (Fuzhou, China) and Hangzhou Lvhe (Hangzhou, China), respectively. PVA (Mw: 89–98 kDa, 99.5%), PEI (60% aqueous solution), sodium sulfate (Na_2_SO_4_, 99.0%), and sodium chloride (NaCl, 99.5%) were supplied by Aldrich (Shanghai, China). Glutaraldehyde (GA, 25 wt% solution in water), carbon black (CB, 99.9%), isopropanol (IPA, AR, ≥99.5%), and n-methylpyrrolidone (NMP, ≥99.0%) were purchased from Meryer (Shanghai, China). Polyvinylidene fluoride (PVDF, Mw = 670–700 kDa, 99%) was provided by Solvay (Atlanta, GA, USA). Hydrochloric acid (HCl, 99.7%) and sodium hydroxide (NaOH, 96%) were supplied by Sinopharm (Shanghai, China). All reagents were of analytical grade and used without further purification.

### 2.2. Electrode Preparation

First, AC (as the main active material), CB (as a conductive agent), and PVDF (as a binder) were mixed in a mass ratio of approximately 8:1:1 and ground thoroughly to achieve uniform mixing. Then, these mixtures were added to NMP and stirred continuously for 12 h to form a viscous and homogeneous slurry. Next, the prepared slurry was coated onto a clean graphite paper using a doctor blade with a controlled thickness to form a sheet electrode. After coating, the electrode was placed in a vacuum-drying oven and dried at 80 °C for 6 h to completely remove the solvent. Finally, the dried electrode was taken out and pressed under a certain pressure to enhance the adhesion between the active material and the current collector, and cut into the desired size to obtain the CDI electrode.

Regarding PDAC, 10 g PEI aqueous solution and 10 g GA were dissolved in 100 mL DI water and stirred uniformly. The mixture was sealed and maintained at 50 °C for 16 h. After that, the resulting product was soaked in 1 L water and stirred at a speed of 50 rpm for 10 min to ensure thorough cleaning. Subsequently, the product was freeze-dried to obtain the insoluble GA-PEI. Next, 1 g GA-PEI, 8 g AC particles, 1 g PVDF, and 1 g CB were added to NMP. The mixture was mechanically stirred for 24 h until all the substances were completely dissolved. Then, the solution was scraped onto graphite paper using a flat scraper. After scraping, the graphite paper coated with the solution was placed in a vacuum-drying oven at 80 °C for 3 h to obtain the final product, denoted as PDAC.

For PVA/PEI membrane-deposited AC (PMAC), specific amounts of dry PVA were dissolved in de-ionized water to prepare an aqueous solution containing 10 wt% PVA. Subsequently, the solution was heated and stirred at 90 °C for 5 h. Next, 10 wt% PEI was introduced into the as-prepared PVA solution. The resulting mixtures were vigorously stirred at room temperature for a continuous period of 24 h to ensure homogeneity. After that, the cast solution was carefully poured onto an AC electrode that was supported by a glass plate. The fabricated electrodes were then dried in an oven at 60 °C for 4 h. Once this drying process was completed, the material was detached from the glass plate and immersed in a crosslinking bath for 1 h at 80 °C. The crosslinking bath was formulated with 90 mL IPA, 10 mL DI water, and 1 mL concentrated HCl, along with 5% GA. Following the crosslinking procedure, the material was again dried at 60 °C for 2 h, and the final product was obtained.

### 2.3. Characterization

Surface morphologies and elemental distributions of AEM, AC, PDAC, and PMAC were characterized by field-emission scanning electron microscopy (FE-SEM, JSM-7800F, JEOL, Tokyo, Japan). Elemental compositions of AEM, AC, PDAC, and PMAC were examined via X-ray photoelectron spectroscopy (XPS, AXIS UltraDLD, Shimadzu, Kyoto, Japan). Water contact angles (WCAs) of AC, PDAC, and PMAC were measured by a contact angle system (Dropmeter A-200, KRUSS, Hamburg, Germany). The functional group distribution of AEM, AC, PDAC, and PMAC was characterized by attenuated total reflectance–Fourier transform infrared spectroscopy (ATR-FTIR, Nicolet 6700, Thermo Fisher Scientific, Waltham, MA, USA) in the scanning range of 400–4000 cm^−1^. The specific surface areas and pore volumes of electrodes were determined using N_2_ adsorption–desorption isotherms (AutosorbIQ3, Quantachrome, Boynton Beach, FL, USA). The tensile strength of the electrodes was measured using an electronic universal testing machine (QJ210, Shanghai, China).

The point of zero charge (pH_pzc_) of AC, PDAC, and PMAC was evaluated by using the solid addition method. Specifically, 0.2 g electrode samples were dispersed into 0.01 M NaCl solutions. The original solution pH (pH_o_) was controlled from 4 to 9. The feed solutions containing electrode samples were kept stable for 48 h, and final pH (pH_f_) values were recorded. The pH_o_ and pH_o_–pH_f_ were set as horizontal and vertical coordinates, respectively, to form a curve, and the intersection was defined as *pH_pzc_*.

### 2.4. System Setup and Experiments

**Figure 1:**
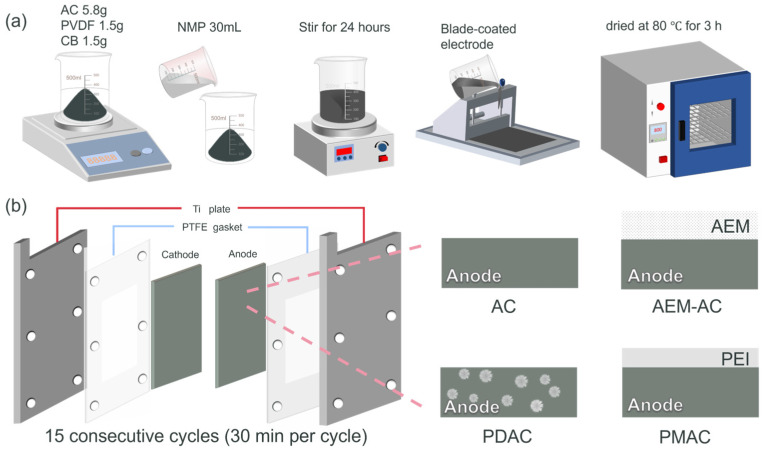
(**a**) Schematic diagram of electrode preparation, (**b**) the experimental setups of CDI with different electrodes.

The whole setup typically included a CDI cell, peristaltic pump, powder supply, and feed tank. A CDI cell typically consisted of two parallel electrodes, and they were separated by a polytetrafluoroethylene (PTFE) spacer with a thickness of 1 mm, as illustrated in Figure 1. The CDI unit employs a mesh spacer with a thickness of 100 μm to form a flow channel with a hydraulic diameter of approximately 200 μm. Under normal operating conditions, the Reynolds number dropped to about 2~10, and this result confirmed that the influent was in the form of laminar flow. During CDI operation, the optimized flow channel still resulted in a dead volume of approximately 20%. Pristine AC was used as a cathode, while AC, AEM-AC, PDAC, and PMAC were used as anodes, respectively. All these systems were operated under the same conditions for better comparison.

Specifically, CDI operated in a batch mode where the influent and effluent were circulated in the same tank with a total volume of 250 mL. During the charging process (25 min), NaCl solution was pumped into the cell at a flow rate of 25 mL/min, and a constant voltage was applied to conduct ion adsorption. The changes in solution conductivity and pH were measured over time using a conductivity meter and pH probe, respectively. The water sample with a volume of 0.5 mL was collected at an interval time for the measurement of chlorine concentration. During the discharging process (5 min), a reversal voltage of 1.0 V was applied to perform ion desorption. To investigate the cycling stability of different systems, 15 consecutive cycles (30 min per cycle) were conducted at 1.2 V, and changes in pH and conductivity were recorded.

The effects of different voltages (0.0, 0.8, 1.2, and 1.8 V) on PMAC performance were explored in treating 250 mg/L NaCl at a pH of 6.5. Next, the initial solution pH was adjusted to 4.0, 6.5, 7.0, and 8.0 to explore the effect of pH on desalination performance in treating 250 mg/L NaCl at 1.2 V. Then, initial NaCl solutions with concentrations of 100, 250, 500, and 700 mg/L were used as feeds to evaluate desalination performance at 1.2 V. Finally, feed solutions with a constant NaCl concentration (5 mM) and varying initial Na_2_SO_4_ concentrations (5, 10, 15, 20 mM) were used to examine the ion-selective adsorption performance.

To clarify the roles of direct anode reaction and H_2_O_2_ oxidation on cycling stability, 15 repeated charge–discharge cycles were performed using 250 mg/L NaCl as a feed solution at 1.2 V with and without dissolved oxygen (DO), respectively. The changes in solution conductivity and pH values were recorded over time. After cycling, the electrodes were dried and the mass was identified. To further compare the stability of AEM-AC and PMAC, 15 repeated charge–discharge cycles were performed using 250 mg/L NaCl as feed solution at 1.2 V.

The salt adsorption capacity (SAC) and charge efficiency (CE) were calculated based on our previous studies [23].

Selectivity between Cl^−^ and SO_4_^2 −^ was quantified by Equation (1) as follows:

(1) $$Selectivity=\frac{Q\int_{t_{0}}^{t_{c}}\left(C_{{{SO}_{4}}^{2-},0}-C_{{{SO}_{4}}^{2-}}\right)dt/\int_{t_{0}}^{t_{c}}C_{{{SO}_{4}}^{2-},0}dt}{Q\int_{t_{0}}^{t_{c}}\left(C_{Cl,0}-C_{Cl}\right)dt/\int_{t_{0}}^{t_{c}}C_{Cl,0}dt}$$

where *C*_SO4_^2−^_,0_, *C*_Cl,0_, *C*_SO4_^2−^, and *C*_Cl_ are the initial SO_4_^2−^, initial Cl^−^, effluent SO_4_^2−^, and effluent Cl^−^ concentrations (mM), respectively, *Q* denotes the volumetric feed flow rate (mL/min), and *t_c_* represents the charging time (min).

The mass loss (%) was calculated by Equation (2) as follows:

(2) $$Mass\;loss=\frac{m_{o}-m_{f}}{m_{o}}$$

where *m_o_* and *m_f_* (mg) represent the original and final mass of electrodes before and after cycling.

## 3. Results and Discussion

### 3.1. Characterization of Electrode and AEM

**Figure 2:**
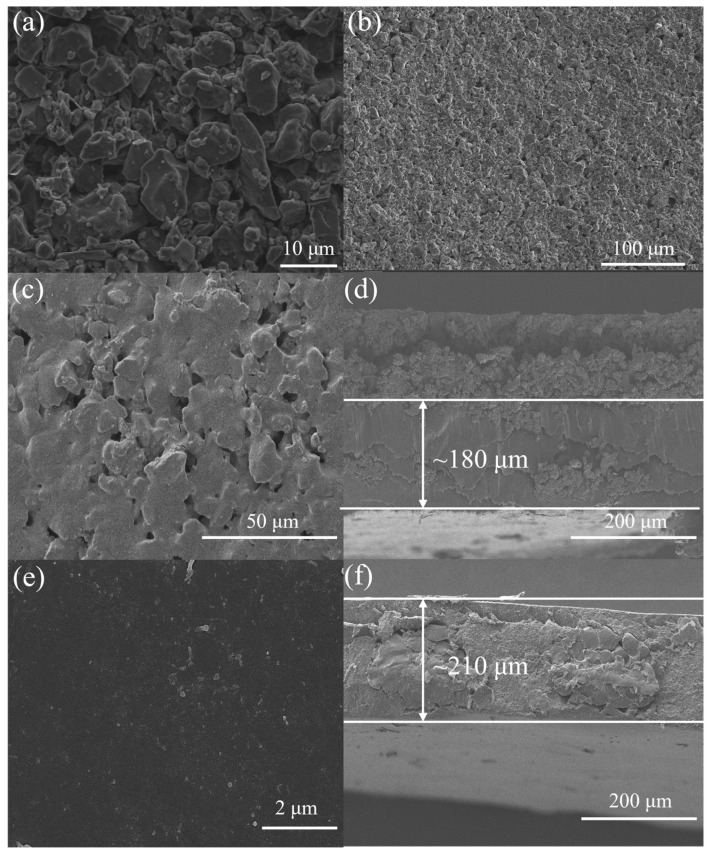
Surface morphologies of (**a**) pristine AC, (**b**) PDAC, (**c**) PMAC, and (**e**) AEM; cross-section morphologies of (**d**) PMAC and (**f**) AEM.

The SEM image, as shown in Figure S1a, indicates that AC particles were irregularly distributed and arranged, with a size distribution of 1–5 μm. As shown in Figure 2a, the surface morphology of the AC electrode and large AC particles with uniform size were distributed on the electrode surface, mixed with small PVDF binders. The particles were tightly bound together by the binders, presenting an irregular, dispersed, and rough structure. A similar structure was also observed for PDAC in Figure 2b. The doped GA-PEI in Figure S1b presented a three-dimensional structure with an average width of approximately 25 μm and a length of approximately 90 μm. For PMAC, depicted in Figure 2c, the available space between AC particles was occupied by polymers with no obvious pore structure observed. From the cross-section image of PMAC, the PVA/PEI membrane with a thickness of approximately 180 μm was deposited on the AC surface. SEM images in Figure 2e,f revealed that AEM with a thickness of 210 μm exhibited a smooth and dense morphology without any detectable cracks, holes, or pores.

In the FTIR result of GA, characteristic absorption peaks typically appeared around 1713 cm^−1^ (C=O stretching of aldehyde groups) and 2820–2720 cm^−1^ (C-H stretching of -CHO groups), confirming the presence of aldehyde functional groups. For PEI, prominent peaks were observed in the region of 3300–3500 cm^−1^ (N-H stretching of amine groups) and 1450–1350 cm^−1^ (C-H bending of alkyl chains), indicating the presence of primary/secondary amine groups and aliphatic backbones. The FTIR of PVA exhibited a strong broad peak around 3200–3600 cm^−1^ (O-H stretching of hydroxyl groups) and a peak near 1087 cm^−1^ (C-O stretching), which were attributed to the signatures of its hydroxyl-functionalized polymeric structure. For PMAC, the broad peak in the region of 3300–3500 cm^−1^ belonged to the stretching vibrations of primary amine (−NH_2_), secondary amine (−NH−), and −OH groups derived from PEI, confirming the successful synthesis of PVA/PEI membrane [24]. The peak located at 2936 cm^−1^ was assigned to the asymmetric and symmetric bands of –CH_2_−. The characteristic peak at 1632 cm^−1^ was attributed to the C=N linkage derived from the crosslinking reaction between the primary amine groups of PEI and CHO groups of GA [25].

**Figure 3:**
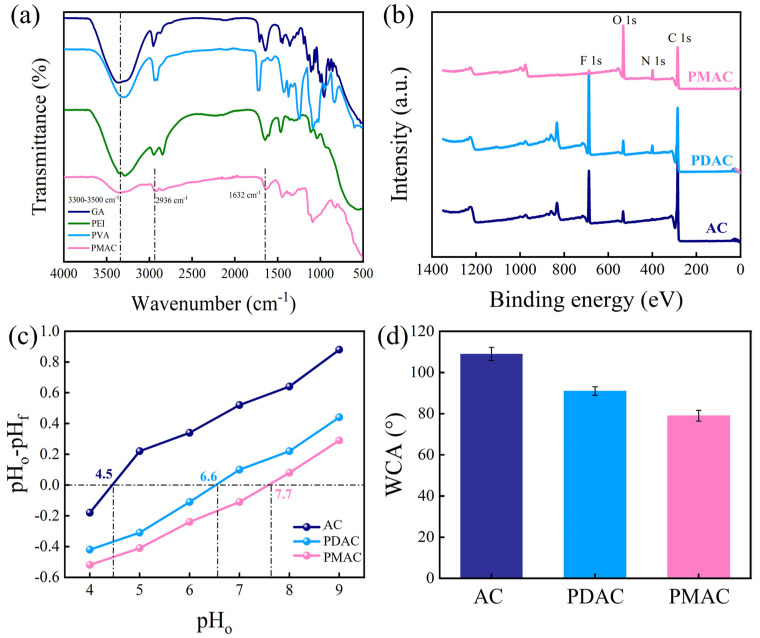
(**a**) FTIR spectrum, (**b**) XPS wide-scan spectrum, (**c**) pH_pzc_, (**d**) WCAs of different electrodes.

Figure 3b depicts the XPS spectrum of different electrodes, and the corresponding element contents are listed in Table 1. AC had obvious peak signals at 285.6, 400.1, 532.6, and 663.3 eV, corresponding to C 1s, N 1s, O 1s, and F 1s with concentrations of 81.7%, 0.9%, 4.6%, and 12.8%, respectively. The appearance of F came from PVDF, where the PVDF binder acted as a physical bridge, embedding AC and CB into a cohesive network through mechanical interlocking. PDAC exhibited similar characteristic peaks, while its N concentration significantly increased to 4.4%, demonstrating the successful doping of GA-PEI. PMAC exhibited characteristic peaks of C 1s, N 1s, and O 1s; its N concentration further increased to 6.5%, owing to the complete exposure of groups on the PVA/PEI membrane. The deconvolution of XPS N1s of PMAC in Figure S2 mainly consisted of −NH_2_, −NH−, and tertiary amines (>N−), which were located at 400.8, 400.1, and 399.5 eV, respectively, and these protonated to yield −NH_3_^+^ groups and acted as an anion-selective polymer [26]. For AEM in Figure S1f, three distinct peaks assigned to C 1s, N 1s, and O 1s were clearly observed.

Figure 3c presents the *pH_pzc_* values of different electrodes at different pH values. The *pH_pzc_* result of AC (4.1) indicated that it was positively charged at pH < 4.1 and became negatively charged at pH > 4.1. Given that the pH of the prepared feed solution was neutral, this negatively charged AC electrode might have weakened the electrostatic interaction when it was used as the anode. The *pH_pzc_* of PDAC increased to 5.8 owing to the existence of positively charged groups derived from PEI, while this increment was mild as a part of PEI was covered by CB and PVDF. In contrast, the *pH_pzc_* of PMAC significantly increased to 7.6 as the resulting −NH_2_ on the deposited PVA/PEI membrane tended to be protonated to −NH_3_^+^ in a neutral solution, contributing to the positive surface charge of the electrode [27].

The surface wettability, as an important factor affecting ion adsorption, was evaluated by WCA, and the results are presented in Figure 3d. The WCA of AC was approximately 109.5° owing to the doping of hydrophobic PVDF binder, suggesting its hydrophobic feature. The WCA of PDAC slightly decreased to 90.7°, which might be attributed to the presence of a substantial number of hydrophilic groups derived from PEI, as well as its loose and porous structure [28]. In contrast, the WCA of PMAC decreased to 78.7°, indicating its hydrophilicity characteristic, which might subsequently promote solution permeation and ion migration to the internal electrode pores. Figure S3 presents the tensile strength comparison of different electrodes. It was found that PMAC exhibited a higher strength of 5.8 MPa than that of AC and PDAC, suggesting that PMAC posed strong resistance to mechanical erosion and exhibited superb durability during long-term operation.

### 3.2. Performance Evaluation of Different Electrodes

**Figure 4:**
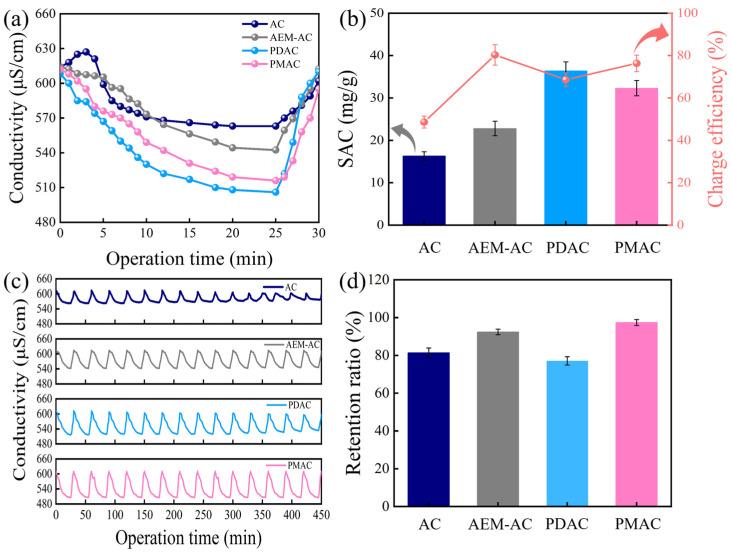
Performance comparison of different electrodes: (**a**) solution conductivity changes with operation time, (**b**) SAC and charge efficiency, (**c**) conductivity changes during 15 cycles of operation, (**d**) retention ratios after 15 cycles of operation.

Figure 4 shows the changes in solution conductivity with operation time during the charging process of different systems. For AC, the solution conductivity increased initially, arising from the desorption of residual ions inside AC pores, which was widely known as the co-ion repulsion effect. After that, as illustrated in Figure S4, the charged ions (Na^+^, Cl^−^) moved toward oppositely charged electrodes and desalination was realized. In contrast, for the other three systems, it was observed that the curves all decreased rapidly initially, owing to the effective adsorption of salts by opposite electrodes, and gradually became stable due to the adsorption equilibrium. After 25 min operation, the solution conductivity of 250 mg/L NaCl decreased from 610 to 563, 542, 508, and 513 μS/cm when AC, AEM-AC, PDAC, and PMAC were used as anodes, respectively. During the discharging process, solution conductivity increased rapidly and recovered to the initial value within 5 min owing to the effective desorption of ions from the electrode to the solution. Corresponding SAC and charge efficiency are presented in Figure 4b; the SAC values followed the order of PDAC (36.3 mg/g) > PMAC (33.4 mg/g) > AEM-AC (22.8 mg/g) > AC (16.3 mg/g), and the charge efficiency followed the order of AEM-AC (80.3%) > PMAC (76.5%) > PDAC (67.8%) > AC (48.4%). The performance of PMAC was further compared with other reported electrode materials, and a summary of electrode performance is presented in Table S1.

Cycling stability is a critical parameter for evaluating practical CDI performance, and the changes in solution conductivity treated by different systems are presented in Figure 4c. It was found that AC and PDAC exhibited relatively stable conductivity variations in the initial cycles, while the amplitude of conductivity variation gradually narrowed, suggesting the deterioration of desalination performance. AEM-AC and PMAC both presented highly stable conductivity changes. More specifically, as illustrated in Figure 4d, SAC decreased to 14.2 mg/g with a retention ratio of 81.4% for AC after 15 cycles of operation, and a more significant reduction in retention ratio to 77.1% was observed for PDAC. In contrast, AEM-AC and PMAC both exhibited highly stable performance with retention of 92.4% and 97.5%, respectively.

The cycling experiments were further prolonged to compare the stability of AEM-AC and PMAC. PMAC maintained a high retention ratio, while the ratio of AEM-AC decreased to 83.3% after 30 cycles of operation, and this discrepancy was mainly attributed to the significant aging of the AEM. Figure S3 shows the surface morphology of the AEM after 30 cycles of operation, after exposure to impurities, microorganisms, and other substances in water; these may adsorb and deposit on the AEM surface, forming protrusions and granular substances, which increase the surface roughness [29]. Additionally, under continuous mechanical stress, such as long-term erosion by water flow or improper operation during membrane component installation or disassembly, the aging membrane surface is prone to scratches, or local depressions are formed in some weak areas, which might change the original surface morphology of the AEM and affect its normal performance, such as a reduction in IEC (1.5 to 1.2 mmol/g) and increment of surface impedance (2.4 to 3.2 Ω/cm^2^). Furthermore, as shown in Figure S6, CDI equipped with PMAC exhibited a lower energy consumption than that of AEM-AC. Overall, given the superb adsorption capacity, low energy consumption, and robust cycling stability, PMAC holds great promise as a CDI anode for water desalination compared to the assembly of the membrane.

### 3.3. Mechanism Investigation

**Figure 5:**
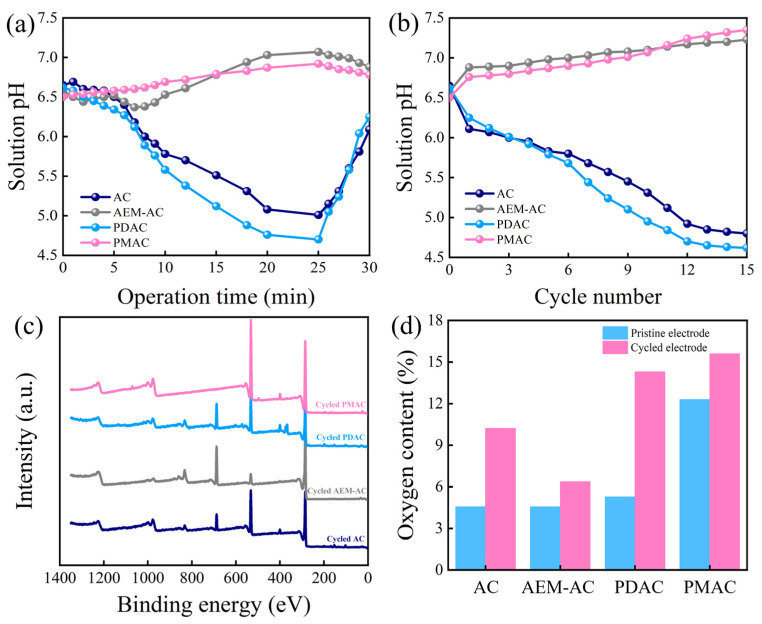
(**a**) Changes in solution pH during charging, (**b**) changes in solution pH during cycling, (**c**) XPS spectrum of different cycled electrodes, (**d**) oxygen content of surface of different electrodes.

**Figure 6:**
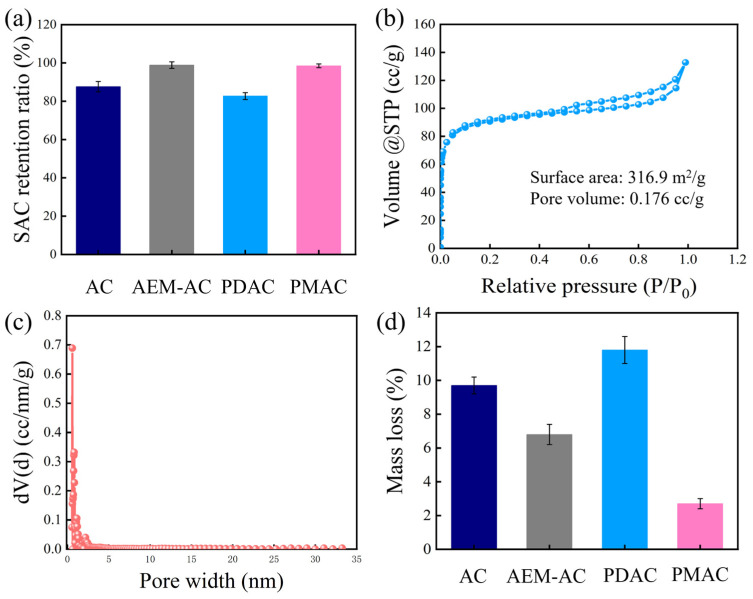
(**a**) SAC retention of CDI with different electrodes after cycling in NaCl solution without DO, (**b**) N2 adsorption–desorption isotherm, (**c**) pore width of PDAC, (**d**) mass loss of different electrodes after 15 cycles of operation.

The mechanisms underlying the performance discrepancies of different systems were investigated via solution pH variations, cycling experiments, and electrode characterizations, with corresponding results shown in Figure 5 and Figure 6. During the charging process, the adsorption of counter-ions from the solution to electrode pores and the repulsion of co-ions from electrode pores into the solution occurred simultaneously, which resulted in a temporary increase in solution conductivity and reduced the charge efficiency. Furthermore, as illustrated in Figure 5a, the solution pH continuously decreased from 6.5 to 5.0 during charging owing to the occurrence of anode oxidation, and this reaction inevitably caused energy loss [23]. These findings revealed that the poor desalination adsorption capacity of AC was mainly attributed to the serious co-ion repulsion and anode oxidation. After 15 cycles of operation, the final solution pH significantly decreased to 4.81 (Figure 5b). Figure 5c,d show the XPS results of different cycled electrodes, and detailed element distributions are supplied in Table 2. Compared to the oxygen content of pristine AC (4.6%), the oxygen content of cycled AC increased remarkably to 14.4%, and this suggested that AC underwent serious oxidation caused by direct anode reaction and H_2_O_2_ oxidation. In particular, it was reported that the anode oxidation of the carbon electrode would itself result in damage to the internal pore structure and increase the interface resistance, subsequently deteriorating performance, as well as being responsible for the terrible cycling stability of AC with an SAC retention ratio of 80.3%. After 15 cycles of operation in treating NaCl without DO, the SAC retention ratio decreased to 87.1% in Figure 6a, suggesting that direct anode reaction was the dominant factor affecting cycling stability.

Comparatively, the existence of a selective AEM in front of the anode prevented co-ions (Na^+^) from exiting into the spacer channel and confined these co-ions in the electrode pores. As co-ions gradually accumulated with a concentration higher than that in the spacer channel, more counter-ions (Cl^−^) would transport through the AEM and be captured by the electrode to compensate for maintaining electroneutrality in the electrode pores. Consequently, the co-ion expulsion was minimized. Meanwhile, the AEM could be isolated inside the AC to contact with the feed and prevent the transport of most H_2_O_2_ inside the AC. The change in solution pH from 6.5 to 6.9 suggests the inhibition of anode oxidation, thus reducing energy loss and leading to performance improvement. After 15 cycles of operation, both the slight increment of oxygen content from 4.6% to 6.5% and the continuous upward trend of solution pH confirmed that the AC underwent mild oxidation owing to the protective role of the AEM. Therefore, AEM-AC showed mild SAC decline with a retention of 92.4% over 15 repeated charge–discharge cycles owing to the H_2_O_2_ oxidation.

When PDAC served as the anode, the abundant positively charged groups from GA-PEI enhanced electrostatic interactions, thereby facilitating the attraction of negatively charged Cl^−^ ions. To maintain the electroneutrality of the solution, a greater amount of Na^+^ ions were consequently captured by the cathode. Meanwhile, PDAC, endowed with a high specific surface area (Figure 6b,c), offered ample accessible sites for ion adsorption, which further boosted the overall ion adsorption capacity. Furthermore, the doping of ion exchange PEI polymers into the AC electrode resulted in the formation of an ion-selective layer lining the electrode pores. This layer effectively inhibited the transport of trapped Na^+^ ions from the electrode pores into the spacer channel, thus minimizing the co-ion effect [30]. Therefore, PDAC exhibited the highest SAC. However, the PEI polymers within the AC also exerted dual effects: they furnished abundant redox-active sites while simultaneously inducing severe anode-side reactions. This is evidenced by a notable reduction in pH to 4.6 and a substantial increase in oxygen content from 5.3% to 17.7% after cycling. Additionally, as presented in Figure 6d, PDAC with low tensile strength experienced significant mass loss (11.8%) during long-term cycling. This phenomenon can be attributed to the weak physisorption of the polymer onto AC particles, which leads to the detachment of PEI from the electrode matrix over repeated cycles. Therefore, PDAC demonstrated poor cycling stability, with a retention of 77.1%, in which the direct anode reaction played a leading role.

When PMAC was used as the anode, the positively charged PEI membrane regulated the movement of Cl^−^ and repelled the transport of Na^+^ from the spacer channel into the AC; meanwhile, this membrane prevented the transport of trapped Na^+^. Thus, the energy was mostly used to adsorb counter-ions. Importantly, compared to the physical attachment of the AEM with a thickness of approximately 210 μm, the thinner PVA/PEI membrane (~180 μm) exhibited a short transport path and reduced electrical resistance, facilitating the transport of Cl^−^ into the electrode pores and subsequent adsorption. Similarly to the role of the AEM, the PEI membrane acted as a barrier to isolate the inside of the AC from contact with the feed and prevented the transport of H_2_O_2_ to the inside of the AC, protecting against damaging Faradaic reactions at the anode surface. Furthermore, a large amount of -OH derived from PVA in its molecular chain formed hydrogen bonds with water molecules, and these hydrogen bonds acted as an antioxidant to interfere with the oxidative corrosion of carbon structure and water [31]. The combination of the membrane layer with AC greatly improved the mechanical strength of the PMAC; hence, the PMAC experienced slight moss loss under continuous mechanical abrasion of the feed with the electrode surface during cycling (Figure 6d). All these factors contributed to the superb desalination performance and robust cycling stability of the PMAC.

### 3.4. Effect of Operation Parameters on PMAC Performance

**Figure 7:**
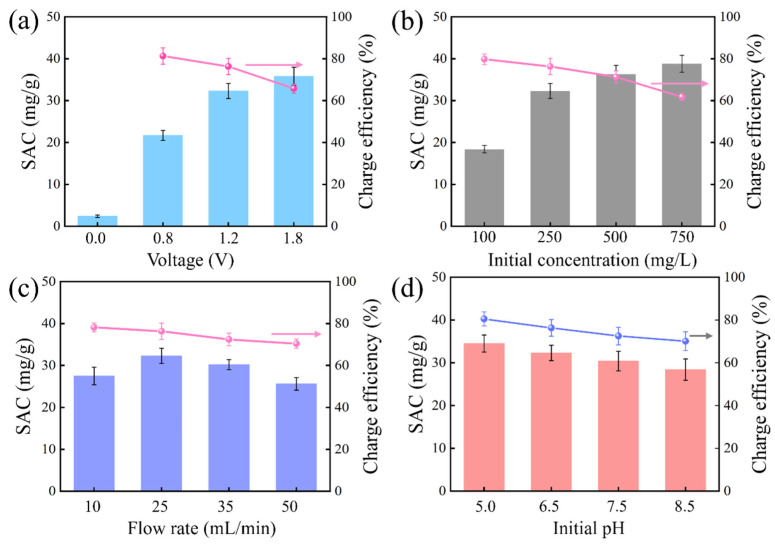
Effects of parameters on PMAC performance: (**a**) applied voltages, (**b**) initial NaCl concentrations, (**c**) flow rates, (**d**) initial pH.

In Figure 7a, CDI performance was highly dependent on the fact that SAC increased with voltage, reaching a maximum value (35.8 mg/g) at 1.8 V. The poor desalination performance at 0 V suggested that ion behavior in CDI was dominated by electrosorption rather than physical adsorption. High voltage produced strong electrical interaction between the two electrodes and facilitated effective ion adsorption by the electrodes. However, the charge efficiency decreased to 65.8% correspondingly as the voltage increased to 1.8 V, which might be attributed to the parasitic side reactions such as water electrolysis and chloride oxidation [32]. Figure 7b shows the desalination performance at different initial feed concentrations at 1.2 V, and it was observed that the SAC increased from 18.4 to 38.5 mg/g when the NaCl concentration increased from 100 to 750 mg/L. An elevated NaCl concentration served to decrease the solution impedance and augment the electric driving force for ion migration, thus promoting ion adsorption. However, there existed abundant co-ions and counter-ions in high-ionic solution, indicating an aggravated co-ion repulsion effect, consistent with the reduction in charge efficiency from 79.7% to 61.6%. Given that CDI was suitable for treating salt solutions with moderate concentrations [15], the performance of AC, AEM-AC, and PDAC was also evaluated in a wide range of solution concentrations. As shown in Figure S4, the SAC followed the order of PDAC>PMAC>AC>AEM-AC when using 100 mg/L NaCl as the feed solution, while it followed the order of PMAC>AEM-AC>PDAC>AC when using 500 and 1500 mg/L NaCl as feed solutions, further confirming the great promise of PMAC in water desalination with a wide range of salt concentrations. In Figure 7c, PMAC demonstrated a superior SAC obtained at a flow rate of 25 mL/min, which decreased at increased flow rates. There was insufficient time for ions to transport through the flow channel to the inside of the pores and subsequent adsorption at a high flow rate. Furthermore, a higher flow rate would lead to high pressure and continuous mechanical abrasion of the electrode surface, exacerbating performance loss. The performance of PMAC was explored at different initial pH values, as presented in Figure 7d. It was found that desalination performance was highly dependent on pH, and the SAC decreased to 28.5 mg/g as pH increased to 8.5. At high pH, abundant OH^−^ existed in aqueous solution and competed in adsorption with Cl^−^. Furthermore, the positive charge density of PMAC decreased as pH increased, weakening the electrostatic attraction between PMAC and Cl^−^, thus resulting in poor performance.

### 3.5. Monovalent Ion Selectivity of PMAC

**Figure 8:**
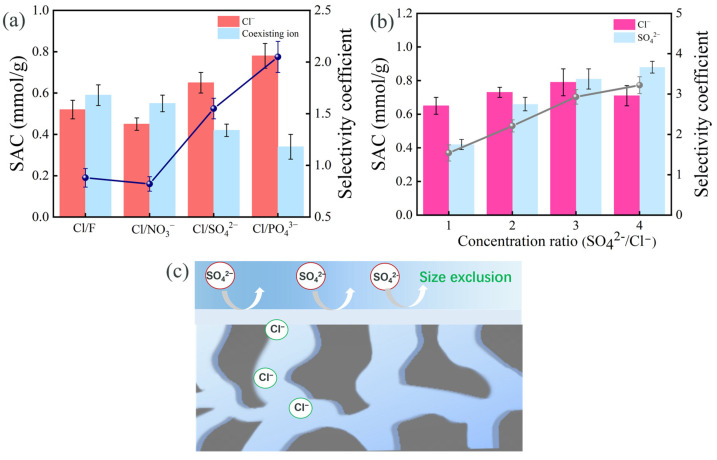
(**a**) Ion selectivity in different mixed solutions, (**b**) ion selectivity in the presence of SO_4_^2−^ with different concentrations, (**c**) ion selectivity mechanism.

The adsorption selectivity of PMAC towards monovalent ions was explored in different mixed solutions with equal concentration. As depicted in Figure 8a, the selectivity coefficients of PMAC towards Cl^−^ were 0.9, 0.8, 1.8, and 2.1 in the presence of NO_3_^−^, F^−^, SO_4_^2−^, and PO_4_^3−^, respectively, confirming the superb monovalent ion selectivity performance. As illustrated in Figure 8c, though divalent ions with high charge could create strong electrostatic interaction with the electrode, the dense PVA/PEI membrane acted as a barrier preventing the transport of these ions with a large hydration ratio and subsequent adsorption by the electrode. Compared to that of SO_4_^2−^ (3.8 Å) and PO_4_^3−^ (3.1 Å), Cl^−^ with a smaller hydration radius exhibited a stronger size affinity and preferentially transported through the dense PVA/PEI membrane [33], leading to the selective adsorption of Cl^−^, confirming that size exclusion dominated the elective adsorption performance of PMAC rather than charge affinity. The adsorption selectivity of PMAC was further investigated in NaCl/Na_2_SO_4_ mixed solutions with different concentration ratios. As seen in Figure 8b, it was observed that in the presence of 5 mM SO_4_^2−^, PMAC demonstrated a higher selectivity over Cl^−^, with a monovalent selectivity coefficient of 1.8. As the SO_4_^2−^ concentration increased to 15 mM, both the SAC and selectivity of PMAC for Cl^−^ increased correspondingly. The high ion concentration decreased the solution impedance and enhanced the driving force for Cl^−^ adsorption. However, the SAC for Cl^−^ decreased when the SO_4_^2−^ concentration increased to 20 mM. In this case, abundant SO_4_^2−^ accumulated on the PEI membrane surface and underwent dehydration to transport through the PEI membrane, leading to its adsorption by PMAC [34]. Given the limited adsorption sites inside PMAC, SO_4_^2−^ with a high concentration compressed the electrical double layer and was preferentially adsorbed by PMAC [35], thus inhibiting the adsorption of Cl^−^. In addition, in the wide-range concentration of SO_4_^2−^, the selectivity coefficient of PMAC toward Cl^−^ was always above 1.0, confirming its superb monovalent ion selectivity.

## 4. Conclusions

In this study, we compared the desalination performance for four different CDI types (AC, AEM-AC, ion exchange polymer-doped AC PDAC, and ion exchange membrane-like deposited AC PMAC), with the aim of clarifying their influences on the ion adsorption and stability. Results showed that AEM, PDAC, and PMAC utilized charge effectively and exhibited higher adsorption capacities than that of AC; however, polymer doped into AC could preserve the AC pore structure and reduce interfacial resistance, endowing PDAC with the best adsorption performance, while it suffered from serious anode oxidation and mass loss, showing poor stability. Similar to the role of the AEM, the direct membrane deposition on AC minimized the co-ion adsorption/desorption, improved the charge utilization, and weakened the anode oxidation. Compared to the physical attachment of the AEM in front of the AC, the integration of the membrane layer with the AC can improve the mechanical strength of PAMCE and inhibit the mass loss during long-term operation; meanwhile, the thin PVA/PEI membrane could shorten the transport path, contributing to the superior adsorption capacity and robust stability. Overall, these findings provide critical insights for the future design and optimization of high-performance CDI electrodes—specifically guiding the development of electrodes balancing high adsorption capacity and long-term stability, which will advance the practical application of CDI as a promising, scalable brackish water desalination technology.

## Figures and Tables

**Table 1:** Element distributions of different electrodes.

Electrode	C (%)	N (%)	O (%)	F (%)
AC	81.7	0.9	4.6	12.8
PDAC	69.8	4.4	5.3	20.5
PMAC	65.4	7.5	24.4	2.7

**Table 2:** Element distributions of different electrodes after cycling.

Electrode	C (%)	N (%)	O (%)	F (%)
Cycled AC	74.6	0.7	14.4	10.3
Cycled AEM-AC	74.3	0.4	6.5	18.8
Cycled PDAC	69.3	4.4	17.7	8.6
Cycled PMAC	67.1	4.3	27.2	1.4

## Data Availability

The original contributions presented in this study are included in the article and Supplementary Material. Further inquiries can be directed to the corresponding authors.

## Supplementary Materials

The following supporting information can be downloaded at: https://www.mdpi.com/article/10.3390/membranes15110338/s1, Figure S1. Surface morphology of (a) AC particles and (b) GA-PEI, (c) FTIR spectrum of GA-PEI, (d) XPS spectrum of GA-PEI, (e) FTIR spectrum of AEM, (f) XPS spectrum of AEM; Figure S2. High-resolution N 1s spectra of PMAC; Figure S3. Tensile strength of AC, PDAC, and PMAC; Figure S4. Ion transport mechanism in CDI; Figure S5. Surface morphology of AEM after 30 cycles of operation; Figure S6. Energy consumption of PMAC and AEM-AC; Figure S7. SAC comparison of electrodes at wide concentrations ranging from 100 to 1500 mg/L. Table S1 Performance comparison of PMAC with other reported electrodes [36,37,38,39,40,41,42].

## Abbreviations

The following abbreviations are used in this manuscript:

Activated carbon	AC
Glutaraldehyde	GA
Polyethyleneimine	PEI
PEI-doped activated carbon	PDAC
PEI-based membrane activated carbon	PMAC
Anion exchange membrane	AEM
Salt adsorption capacity	SAC
Capacitive de-ionization	CDI
Water contact angle	WCA
