# Supplementary material for: Ion Exchange Membrane-like Deposited Electrodes for Capacitive De-Ionization: Performance Evaluation and Mechanism Study
Source: Membranes (Basel). 2025 Nov 11;15(11):338. doi: 10.3390/membranes15110338 (PMC12654372; doi:10.3390/membranes15110338)
Supplement: Supplementary file 1 [file membranes-15-00338-s001.zip › membranes-3952919-supplementary.pdf]

Supplementary Information

Ion Exchange Membrane-Like Deposited Electrodes for Capacitive Deionization: Performance Evaluation and Mechanism Study

Siyue Xue[a,b], Chengyi Wang[a], Tianxiao Leng[a], Chenglin Zhang[a], Long-Fei Ren[a,b,c,d,*], Jiahui Shao [a,b,*]

[a]*State Key Laboratory of Green Papermaking and Resource Recycling, School of Environmental Science and Engineering, Shanghai Jiao Tong University, Shanghai 200240, Shanghai, PR China.*

[b]*National Observation and Research Station of Erhai Lake Ecosystem in Yunnan, Shanghai Jiao Tong University Yunnan Dali Research Institute, Dali 671006, Yunnan, PR China.*

[c]*Yazhou Bay Institute of Deepsea Science and Technology, Hainan Research Institute, Shanghai Jiao Tong University, Hainan, PR China.*

[d]*Chongqing, Research Institute, Shanghai Jiao Tong University, Chongqing, PR China.*

**Correspondence to:*
Long-Fei Ren, E-mail: longfeiren@sjtu.edu.cn.
Jiahui Shao, E-mail: jhshao@sjtu.edu.cn.

A manuscript prepared for possible publication in

Membranes

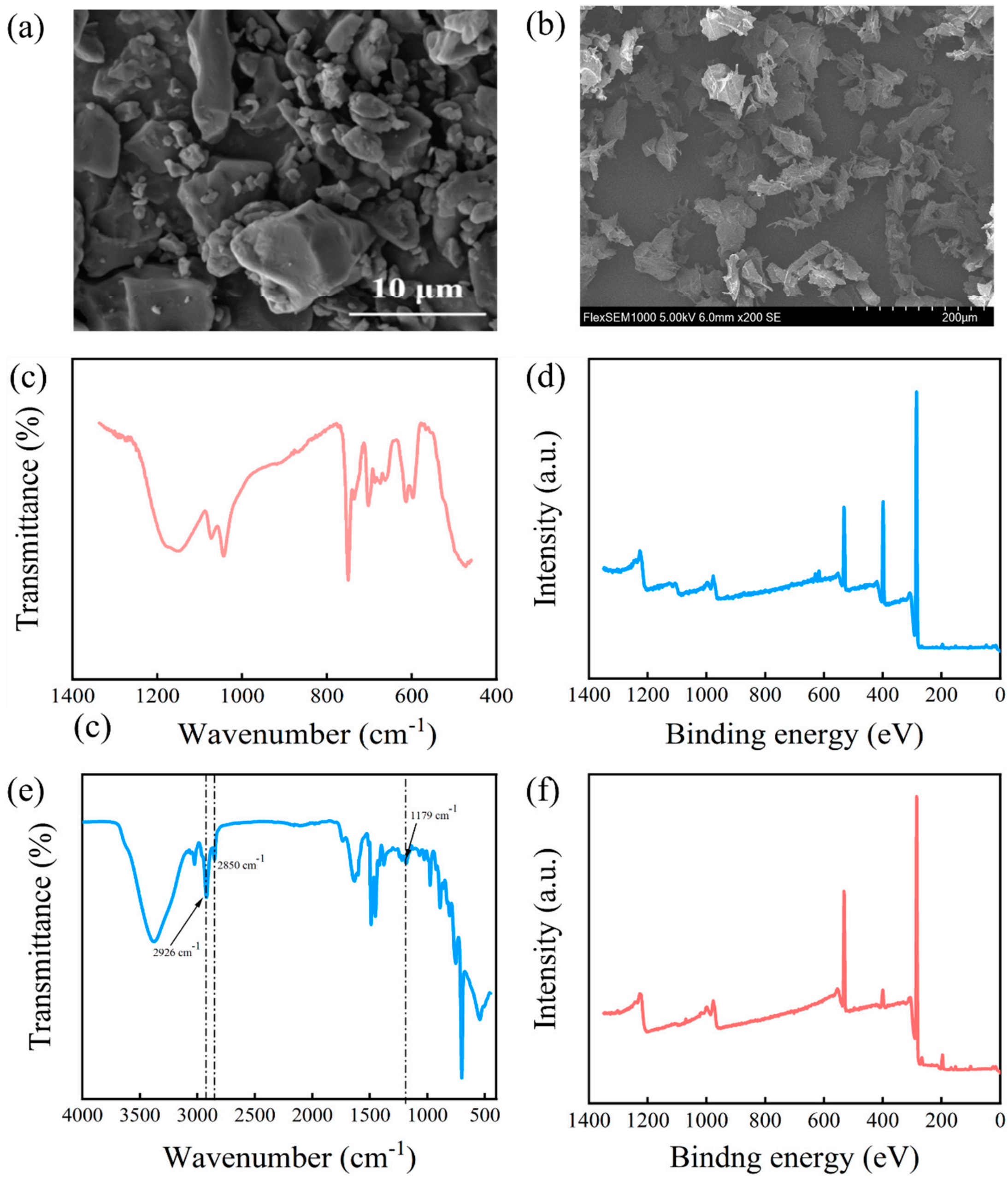

Figure S1. Surface morphology of (a) AC particles and (b) GA-PEI, (c) FTIR spectrum of GA-PEI, (d) XPS spectrum of GA-PEI, (e) FTIR spectrum of AEM, (f) XPS spectrum of AEM.

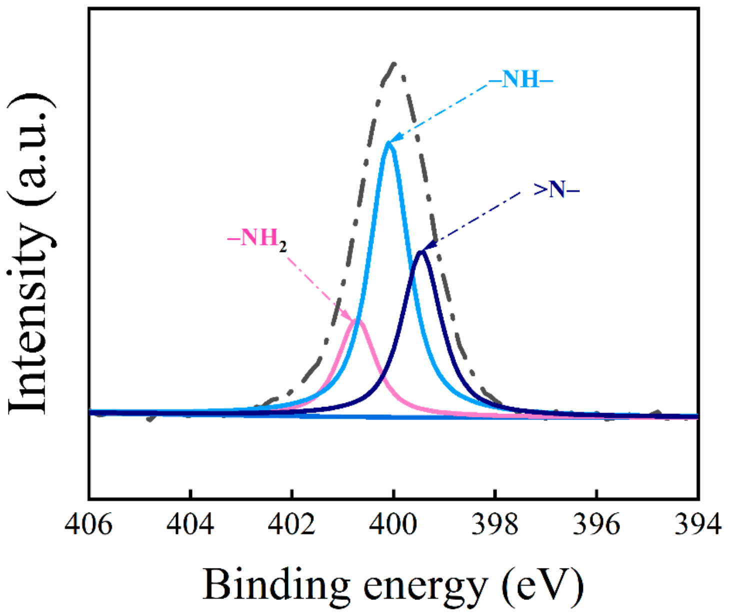

Figure S2. High-resolution N 1s spectra of PMAC.

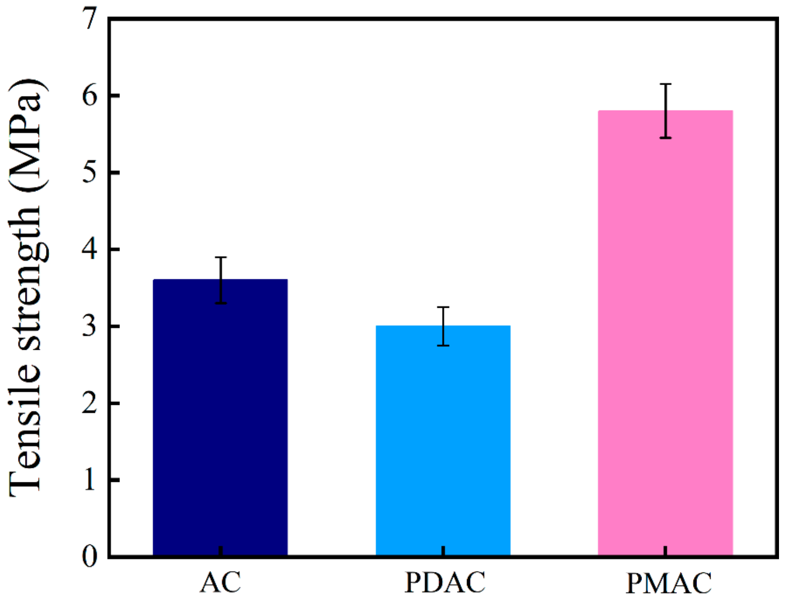

Figure S3. Tensile strength of AC, PDAC, and PMAC.

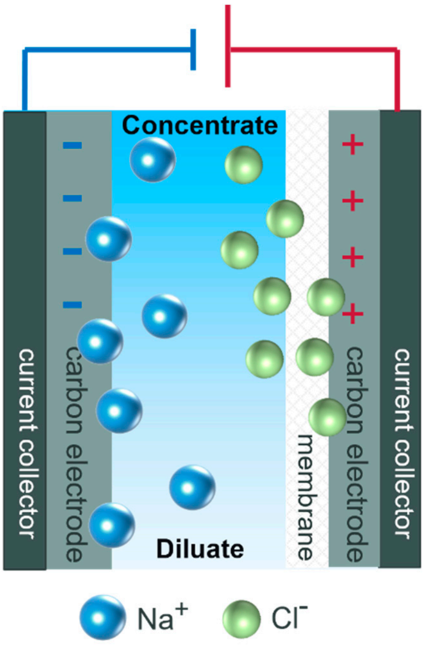

Figure S4. Ion transport mechanism in CDI.

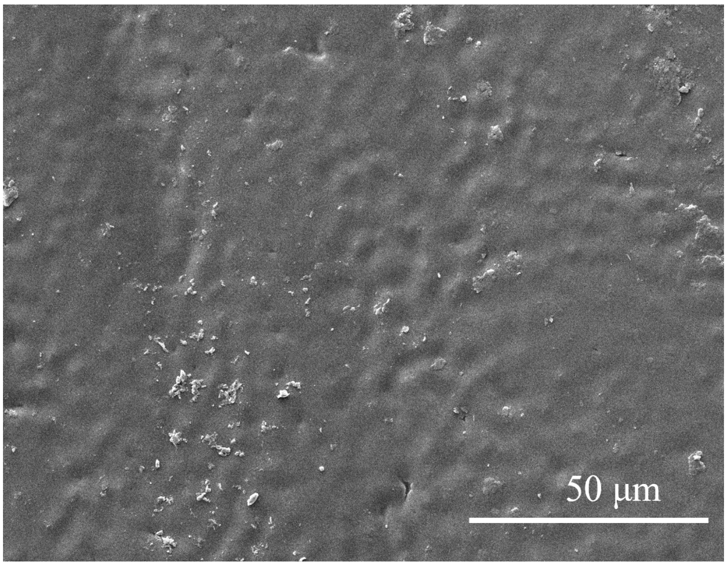

Figure S5. Surface morphology of AEM after 30 cycles of operation.

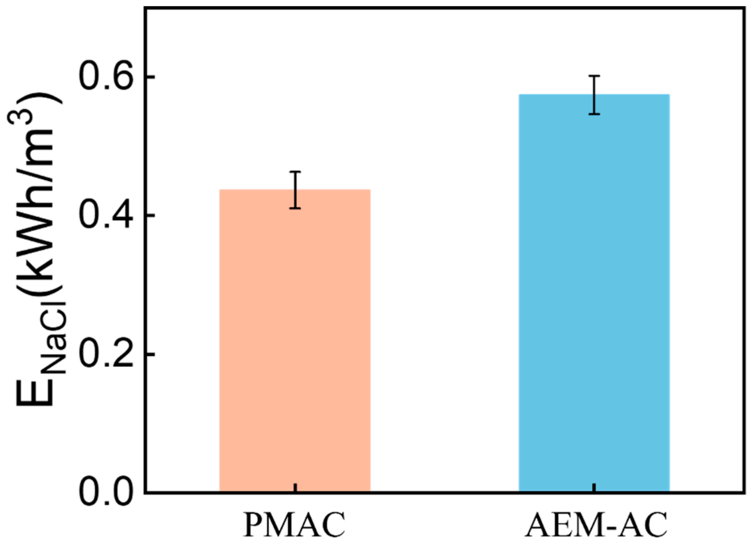

Figure S6. Energy consumption of PMAC and AEM-AC.

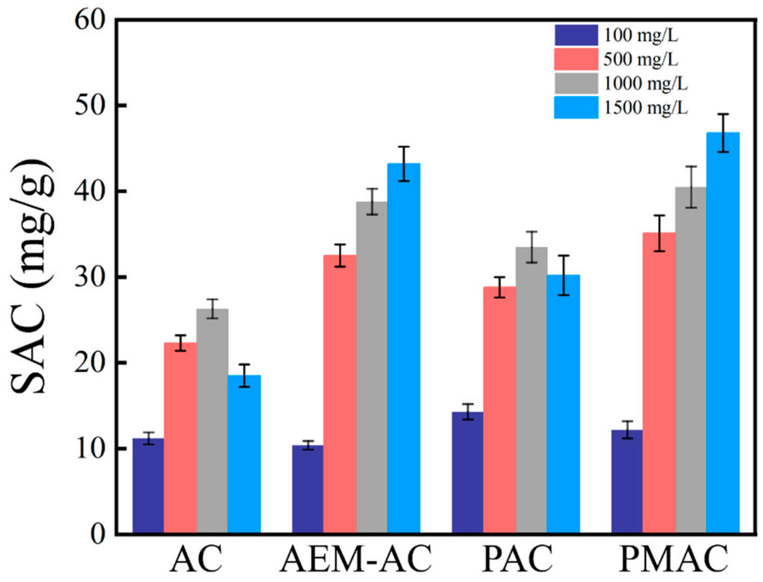

Figure S7. SAC comparison of electrodes at wide concentrations ranging from 100 to 1500 mg/L.

Table S1. Performance comparison of PMAC with other reported electrodes

Electrode	NaCl concentration (mg/L)	SAC (mg/g)	Voltage (V)	Ref.
MnO_2/MXene	1000	30.5	1.2	[36]
$Ti_3C_2T_x$/C	500	24.3	1.2	[37]
AC@$Si0_2$	300	17.0	1.2	[38]
AMX/Speek-80	584.4	23.8	1.0	[39]
CMGO	400	22.5	1.4	[40]
MoS_2/MXene	500	23.9	1.2	[41]
IEP-CCE	1000	20.3	1.5	[42]
PMAC	250	33.4	1.2	This work
